# Hepatocellular carcinoma in Native South Asian Pakistani population; trends, clinico-pathological characteristics & differences in viral marker negative & viral-hepatocellular carcinoma

**DOI:** 10.1186/1756-0500-6-137

**Published:** 2013-04-08

**Authors:** Amna Subhan Butt, Saeed Hamid, Ashfaq Ahmad Wadalawala, Mariam Ghufran, Ammar Asrar Javed, Omer Farooq, Bilal Ahmed, Tanveer Ul Haq, Wasim Jafri

**Affiliations:** 1 Section of Gastroenterology, Department of Medicine, The Aga Khan University & Hospital, Stadium Road, Karachi 74800, Pakistan; 2 Department of Family Medicine, The Aga Khan University, Karachi, Pakistan; 3 The Aga Khan University, Karachi, Pakistan; 4 Department of Medicine, The Aga Khan University, Karachi, Pakistan; 5 Department of Radiology, The Aga Khan University, Karachi, Pakistan

**Keywords:** Hepatocellular Carcinoma, Viral marker negative HCC, Viral-HCC, Hepatitis B, Hepatitis C, Pakistan

## Abstract

**Background:**

HCC is the fifth most common cancer globally. Our study was conducted to (1)investigate the trends and clinico-pathological characteristics of Hepatocellular carcinoma among native South Asian patients in Pakistan, (2)to estimate the prevalence as well as the trends of viral marker negative HCC and (3) to compare the clinico-pathological, radiological characteristics, applicability of treatment at diagnosis and prognostic factors among patients with both viral marker negative and viral marker positive-HCC being consulted at Aga Khan University Hospital(AKUH), Karachi, Pakistan.

**Method:**

Patients ≥18 years, already diagnosed to have HCC and visiting AKUH during 1999–2009 were identified using ICD code 1550. The diagnosis of HCC was made in the presence of characteristic features of HCC on triple-phasic CT scan/MRI or with histological findings on biopsy.

**Results:**

645 patients were enrolled. Of these 546(84.7%) were viral-HCC and 99(15.3%) were viral marker negative HCC. Among viral-HCC group underlying etiology of cirrhosis was HCV in 67.9%, HBV in 21.8% and concomitant HBV with HCV or HDV in 10.3% cases. Majority (62.8%) patients had advanced HCC. Larger tumor size (p < 0.001), shorter duration between diagnosis of cirrhosis and HCC (p 0.03), concomitant Diabetes Mellitus (p < 0.001) were found significant factors associated with viral marker negative HCC.

**Conclusion:**

The burden of hepatocellular carcinoma is rising among native South Asian Pakistani population and the viral marker negative HCC are not uncommon in our population. Viral marker negative HCC tend not to be under surveillance as compared to viral-HCC and are diagnosed mostly at advanced stage & when they became symptomatic.

## Background

Despite advancements in the management of cirrhosis, the prevalence of hepatocellular carcinoma (HCC) is on the rise [1]. The prevalence of HCC varies geographically and currently HCC is the fifth most common cancer representing almost 6% of all newly diagnosed cancers globally [2]. It is also one of the common causes of mortality in patients with cirrhosis. Up to 80% of HCC has been reported from South-East Asia and Africa [2,3]. Hepatitis B (HBV) and C (HCV) are the most important risk factors predisposing a patient to having HCC, particularly in Asia [4,5]. However, several patients develop HCC due to the causes other than hepatitis B and C. Therefore, patients who have negative serological markers for hepatitis B and C grouped as viral marker negative or non-B non-C hepatocellular carcinoma (NBNC-HCC). The reported prevalence of NBNC-HCC ranged between 5-15% [5-7]. The viral marker negative HCC is attributable to alcoholic liver injury, autoimmune and metabolic liver diseases, exposure to environmental toxins like aflatoxins B1 and nonalcoholic steatohepatitis (NASH) (5). Since diverse oncogenic pathways are involved, difference exists in natural course and outcome of HCC in these two groups [8-10]. Hence, considering the fact that HBV has a more direct carcinogenic activity, most of HBV related HCC, present at an earlier stage of cirrhosis, with more aggressive tumors as compared to HCV related HCC [11,12]. Likewise it is well expected that the difference exist in natural history and prognosis of HCC due to viral and non-viral etiologies [3,13]. Nonetheless, one should not ignore the fact that there have been few reports regarding viral marker negative HCC which are mostly based upon unselected or post-surgical patients without stratification according to age, liver function, tumor stage and modality of cancer diagnosis [12,14]. Furthermore, the data regarding HCC due to HBV or HCV or hepatitis D (HDV) co-infection is scanty [2]. Therefore, it is of significance that this issue be addressed on a broader scale.

Pakistan is located in the region that is known to have intermediate prevalence for HCV and HBV [2]. However, the data available in Pakistan on HCC is limited and mainly encompasses experiences from a single center with a small sample size and largely emphasizing on viral-HCC. Based upon these results, HCV is the leading cause of HCC in Pakistan followed by HBV [2,15-18]. Furthermore, no data is available on prevalence and prognostic factors of viral marker negative hepatocellular carcinoma in Pakistan. Hence, this study was conducted to (1) investigate the trends and clinico-pathological characteristics of Hepatocellular carcinoma among native South Asian Pakistani patients, and (2) to estimate the prevalence and trends of viral marker negative HCC and (3) to compare the clinico-pathological, radiological characteristics, stage of HCC, applicability of treatment at diagnosis and prognostic factors among native South Asian patients in Pakistan with viral marker negative and viral-HCC being consulted at The Aga Khan University Hospital (AKUH), Karachi, Pakistan.

## Methods

### Study population and duration

This was a retrospective cross sectional study. Patients ≥ 18 years, diagnosed to have HCC, visiting the Gastroenterology ward and clinics, of AKUH from January 1999 to December 2009 were identified from our data base by using ICD code 1550. AKUH is a 563 bedded, largest tertiary care hospital in the metropolitan city of Karachi with a population of 18 million [7]. The medical record coders at AKUH assign numerical codes for diseases and procedures to all records in accordance with standards outlined in the International Classification of Diseases (ICD-9-CM) code book. The information was recorded on demographics, etiology of underlying chronic liver disease, clinical, radiological, histological characteristics and stage of HCC at the time of diagnosis and the initial treatment provided. Cases with any of this missing information were excluded.

### Diagnosis of HCC and cirrhosis

The diagnosis of HCC was made with characteristic features of HCC on triple-phase computerized tomography (CT) scan/magnetic resonance imaging (MRI) or when the concurrent results were found on CT scan/MRI, in the presence of background chronic liver disease, with or without histological verification [1,19]. The presence of arterial enhancement, followed by washout of contrast on porto-venous and delayed phase were considered as typical characteristic features of HCC [1,19]. The diagnosis of cirrhosis was made either on liver biopsy or, in the absence of liver biopsy by clinical and laboratory features of portal hypertension i.e. varices on upper gastrointestinal endoscopy, radiological features suggestive of cirrhosis including nodular liver margins, dilated portal vein, spleenomegaly and ascites [20,21]. The Child-Pugh classification was used to define the severity of liver dysfunction [22]. Modality of HCC diagnosis was defined (i) under surveillance, when it was detected during regular semiannual screening by ultrasound and alfa fetoprotein (AFP), (ii) incidental, when an asymptomatic HCC was discovered outside any surveillance program or during diagnostic procedures performed for some other disease, (iii) symptomatic when HCC was diagnosed during workup after symptom appearance.

### Staging and classification of HCC

The HCC was considered as “nonadvanced” (if the lesion was solitary, ≤5 cm or paucifocal ≤3 lesions, with the largest diameter ≤3 cm, in the absence of vascular invasion and distant metastases) and “advanced,” when the tumor exceeded these limits. Okuda classification was also used to stage HCC [23]. Moreover, the HCC was also classified according to macroscopic types as (i) solitary, (ii) paucifocal (≤3 nodules), (iii) multifocal (>3 nodules), (iv) infiltrative (infiltrating pattern of HCC) or massive (huge mass with a diameter of > 10 cm and an undefined boundaries) [24]. In the presence of ≥2 lesions, the largest tumor was considered as the representative HCC and the diameter of the representative tumor was measured in its greatest dimension and recorded as tumor size.

The tumor size was divided into three groups; (i) <5 cm (ii) 5-10 cm and (iii) > 10 cm. Furthermore, information was recorded regarding hepatic lobes involved; presence of portal vein thrombosis and extra hepatic spread. The Milan criteria were used to define the stage of HCC while evaluating for liver transplantation [19,25]. Moreover, allocation of various treatment options were according to the Barcelona-Clinic Liver Cancer (BCLC) staging classification [26].

### Laboratory parameters

Liver function tests i.e. total bilirubin, serum alanine aminotransferase (ALT), serum albumin, prothrombin time (PT) and serum alpha-fetoprotein (AFP) were recorded. Viral markers for Hepatitis B (HBsAg) and C (anti-HCV antibody) were tested by radioimmunoassay or enzyme-linked immunosorbent assay (ELISA).

Moreover, information was collected regarding co-morbid conditions i.e. Diabetes Mellitus (DM); hypertension; dyslipidemia; complications related to cirrhosis; histological findings on liver biopsy and initial treatment provided for HCC.

### Main outcome measures and potential prognostic factors

We investigated the trends of HCC during the aforementioned time period. The patients were divided into two groups i.e. i) Viral marker negative HCC (those who have undetectable viral markers for both hepatitis B and C i.e. HBsAg and Anti-HCV antibody) and ii) Viral-HCC or viral marker positive HCC (in whom hepatitis B or C or D was detected by HBsAg, anti-HCV antibody or anti-HDV antibody). Patient’s demographic features, clinical, biochemical, histological, radiological characteristics, stage of HCC and applicability of initial treatment provided, were compared between these two groups. Clinical, laboratory parameters compared include demographic characteristics, body mass index (BMI), co-morbid conditions, duration from diagnosis of CLD to diagnosis of HCC, complications due to cirrhosis, Child-Turcotte-Pugh (CTP) score and class, Okuda class, mode of diagnosis, AFP, total bilirubin, ALT, PT, platelets count at baseline, histopathology, radiological features of HCC (location, size/median diameter, number of lesions), macroscopic types, HCC stage, vascular invasion, extra hepatic metastasis and treatment provided were compared as potential prognostic factors between the two groups.

The study was conducted by maintaining compliance with the Helsinki Declaration and was approved by the Ethical review committee of AKUH.

### Statistical analysis

Data was entered and analyzed using SPSS version 17.0. Mean ± SD, ranges were calculated for continuous variables and proportions for categorical variables. To see the trends of HCC, year wise frequencies of HCC cases were also calculated. To see the difference between the two groups independent student *t*-test, chi square and Fisher exact were used where appropriate. Continuous variables were checked for their linearity, by doing quartile and Box Tidwell analysis. Dummy variables were created for variables with more than two categories and the reference group for each variable was defined as the category with the minimal risk for HCC using previous studies. Multi-colinearity was checked among all the independent variables. A univariate logistic regression analysis was conducted to assess the (crude) association of the prognostic factors for viral-HCC vs. viral marker negative HCC. Biological significance and a value of p ≤ 0.25 were considered as criteria for a variable to be significant at univariate analysis. Biological plausible interactions among variables and confounding were also checked. The variables found significant on univariate analysis were included in multivariable logistic regression analysis. Multivariable logistic regression was done and results expressed as odds ratios, along with 95% confidence intervals.

Later, multinomial logistic regression was done [27]; this analysis allowed for a reference category (HCV) to be compared with other categories (HBV, HBV/HCV/HDV co-infection and viral marker negative HCC). This was used to assess the influence of several independent factors, as well as to study the effects of specific variables controlled by confounders. Biological significance and a value of p ≤ 0.25 were considered as criteria for a variable to be significant at univariate analysis. P value of < 0.05 was considered significant and multinomial odds ratios (mOR) were calculated.

## Results

### Trends and clinic-pathological characteristics of HCC

**Figure 1:**
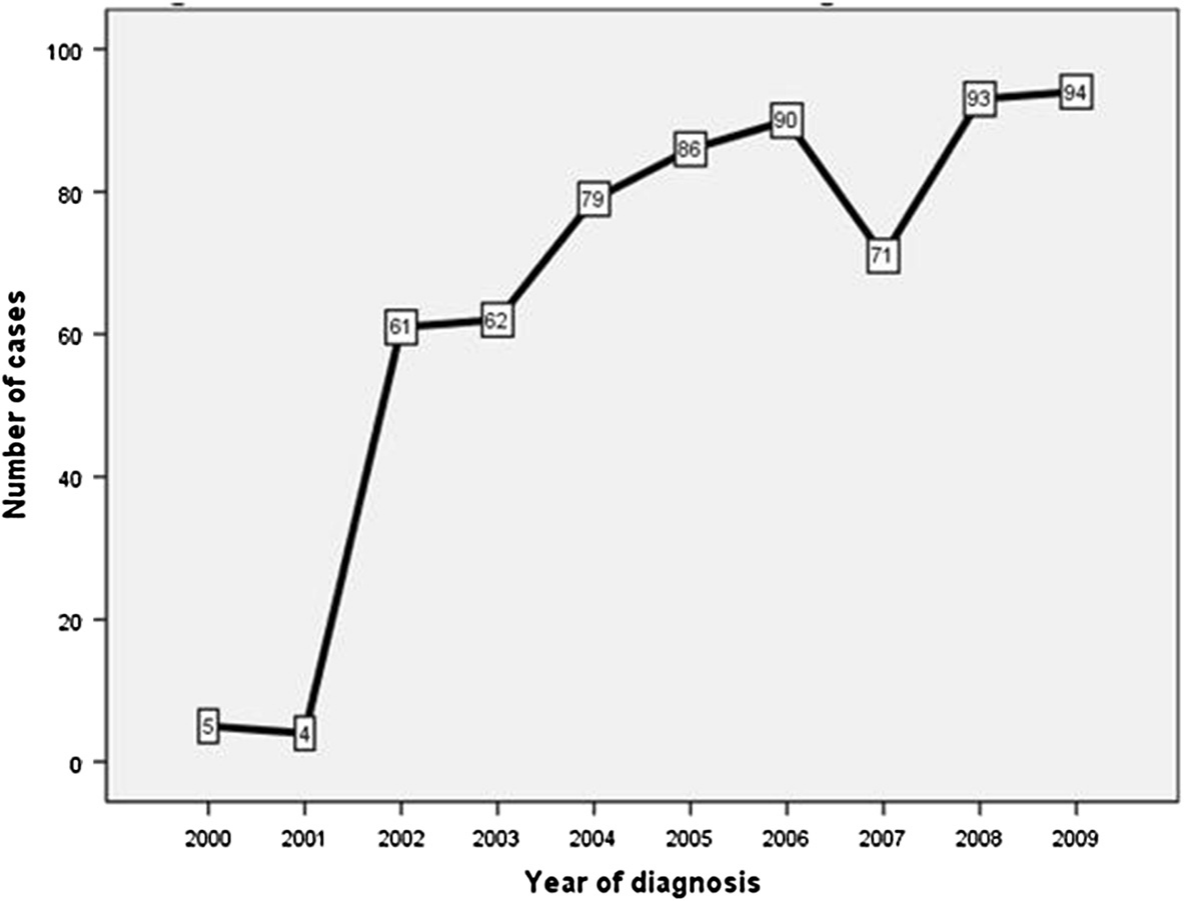
Year wise trends of HCC cases during 1999 till 2009.

**Figure 2:**
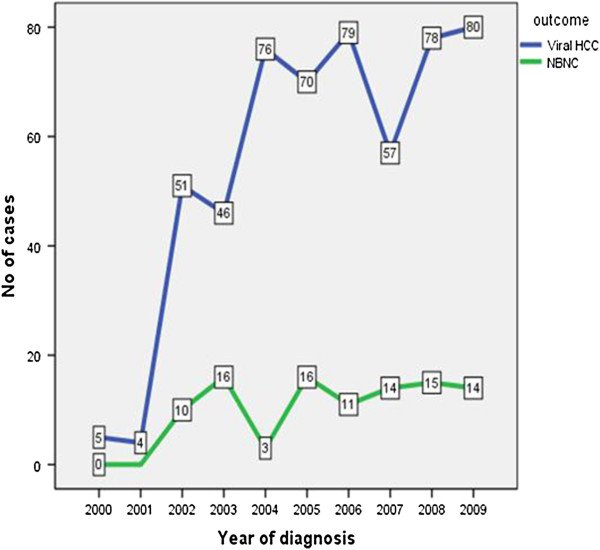
Distribution of viral-HCC and viral marker negative HCC during 1999–2009.

A total of 700 consecutive patients with HCC visited AKUH during 1999 and 2009. Out of these 700 patients, 645 (92%) fulfilled the eligibility criteria and were studied. During the entire study period, an almost consistent increase in number of all HCC cases as well as the viral marker negative HCC cases diagnosed was observed (Figure 1 and 2). The demographic and clinico-pathological characteristics of all HCC patients were as described in Table 1. Majority (70.9%) of our subjects were males. Mean age at inclusion was 56.93 ± 11.15 years (range 18–95 years). There were 546 (84.7%) viral-HCC and 99 (15.3%) viral marker negative HCC. Among viral-HCC group the underlying etiology of cirrhosis was HCV in majority (57.3%) of the cases followed by HBV (18.4%). Concomitant hepatitis B and D or hepatitis B and C were found in 8.7% of all HCC cases. Mean BMI was 24.55 ± 4.39 Kg/m^2^. None among all cases were found as heavy drinkers (i.e. alcohol consumption more than 40 gr/day).

**Table 1:** Demographic and clinical characteristics of all HCC patients and comparison of viral-HCC vs. viral marker negative HCC

	**ALL HCC cases**	**Comparison of viral-HCC vs. viral marker negative HCC mean ± SD/%**		
	**n = (645), mean ± SD/%**			
		**Viral-HCC (n = 546)**	**Viral marker negative HCC (n = 99)**	**P value**
**Age(years)**	56.93 ± 11.15	56.46 ± 10.85	59.57 ± 12.45	**0.01**
**Gender**				
Female	29.1	29.9	25.3	0.34
Male	70.9	70.1	74.7	
**BMI** (Kg/m^2^)				
<18	6.2	5.5	10.1	
18-22.9	28.1	28.6	25.3	0.24
23-24.9	23.6	22.9	27.3	
≥25	42.2	43.0	37.4	
**Diabetes Mellitus**				
No	59.8	61.7	49.5	**0.02**
Yes	40.2	38.3	50.5	
**Hypertension**				
No	65.6	66.8	58.6	0.11
Yes	34.4	33.2	41.4	
**Dyslipidemia**				
No	97.5	97.4	98.0	0.74
Yes	2.5	2.6	2.0	
**Child score**	9.29 ± 2.31	9.27 ± 2.29	9.40 ± 2.43	0.59
**Child class**				
A	12.6	12.8	11.1	0.50
B	42.3	43.0	38.4	
C	45.1	44.1	50.5	
**Okuda class**				
I	14.3	14.3	14.1	0.97
II	61.4	61.5	60.6	
III	24.3	24.2	25.3	
**Complication of CLD**				
No	25.6	26.2	22.2	0.39
Yes	74.4	73.8	77.8	
**Hyperspleenism**				
No	37.8	36.6	44.4	0.14
Yes	62.2	63.4	55.6	
**Ascites**				
No	31.5	31.3	32.3	0.84
Yes	68.5	68.7	67.7	
**PSE**				
No	65.9	66.8	60.6	0.23
Yes	34.1	33.2	39.4	
**Esophageal varices**				
No	47.0	46.3	50.5	0.44
Yes	53.0	53.7	49.5	
**Upper GI bleed**				
No	59.5	59.2	61.6	0.64
Yes	40.5	40.8	38.4	
**Hepatohydrothorax**				
No	91.0	91.4	88.9	0.43
Yes	9.0	8.6	11.1	
**Hepatorenal syndrome**				
No	77.4	77.8	74.7	0.50
Yes	22.6	22.2	25.3	
**Hepatopulmonary syndrome**				
No	92.2	92.9	88.9	0.19
Yes	7.8	7.1	11.1	
**Total Bilirubin(mg/dl)**				
<2	50.2	50.7	47.5	0.48
2-3	20.5	20.9	18.2	
>3	29.3	28.4	34.3	
**Albumin(mg/dl)**				
>3.5	8.1	7.7	10.1	0.73
2.8-3.5	29.3	29.5	28.3	
<2.8	62.6	62.8	61.6	
**ALT (IU/ml)**				
Normal	30.23	28.4	40.4	0.19
Elevated	69.77	71.6	59.6	
**Prothrombin time(seconds)**				
No	92.2	92.9	88.9	0.19
Yes	7.8	7.1	11.1	
**Total Bilirubin(mg/dl)**				
<2	50.2	50.7	47.5	0.48
2-3	20.5	20.9	18.2	
>3	29.3	28.4	34.3	
**Albumin(mg/dl)**				
>3.5	8.1	7.7	10.1	0.73
2.8-3.5	29.3	29.5	28.3	
<2.8	62.6	62.8	61.6	
**ALT (IU/ml)**				
Normal	30.23	28.4	40.4	0.19
Elevated	69.77	71.6	59.6	
**Prothrombin time(seconds)**				
<3	42.8	41.6	49.5	0.14
3-6	34.6	36.1	26.3	
>6	22.6	22.3	24.2	
**Platelets counts(10**^**9**^**/L)**	165.91 ± 107.02	155.72 ± 96.33	222.12 ± 141.01	**<0.001**
**AFP(IU/ml)**				
≤200	58.8	58.2	61.6	0.30
>200	41.2	41.8	38.4	

Majority of our patients had a Child’s class B or C cirrhosis. Almost two third (74.4%) of all patients experienced at least one complication related to cirrhosis before their index presentation. Concomitant diabetes, hypertension or dyslipidemia was found in 40.2%, 34.4% and 2.5% of the cases, respectively. Most of the HCC cases (82.9%) were diagnosed when they were symptomatic, while only 8.2% HCC cases were diagnosed on screening. The duration between diagnosis of chronic liver disease and HCC was 24.01 ± 38.05 months (range 0–195 months). Moreover, majority of patients (62.8%) had advanced HCC. The average maximum tumor size was 5.62 ± 3.67 cm.

### Comparison of viral-HCC and viral marker negative HCC

We compared the demographic and clinical characteristics of patients with viral-HCC and viral marker negative HCC (Table 1). Patients with viral marker negative HCC were significantly older (p 0.01) and almost half of them were affected by concomitant DM (50.5% vs. 38.3%, p 0.02) as compared to viral-HCC. No difference was found in distribution of gender, BMI, severity and complications of cirrhosis, associated co-morbid conditions, biochemical profile and AFP levels. However, patients with viral HCC were more thrombocytopenic than viral marker negative HCC on presentation (p <0.001).

There was no difference in proportion of patients diagnosed with HCC when symptomatic in viral-HCC and viral marker negative HCC group (82.4% vs. 85.9%). Overall only 8.2% HCC cases were diagnosed on screening. However, in contrary to viral-HCC group, small number of patients with viral marker negative HCC were under surveillance (1% vs. 9.5%, p 0.001). It’s not correct to compare HCC in followed patients with those who were not under surveillance for HCC. However, as we found a very small proportion of patients with HCC who were under surveillance, we felt better to see the difference among both group. In 65.7% of non-viral HCC and 41.4% of viral HCC diagnosis of cirrhosis and HCC were made simultaneously. Hence, the duration between diagnosis of cirrhosis and HCC was significantly shorter in viral marker negative HCC group as compared to viral-HCC (p <0.001). This was probably due to the fact that a greater proportion of patients with non-viral HCC was not symptomatic and was not under surveillance for HCC as compared to viral HCC cases. Not only the tumor size was significantly larger (p <0.001), but HCCs were more advanced at presentation in viral marker negative HCC group as compared to viral-HCC (81.8% vs. 59.3%, p < 0.001). No difference was found in the involvement of hepatic lobes, presence of PVT and extra-hepatic spread among both the groups (Table 2).

**Table 2:** Characteristics of HCC and comparison of tumor characteristics among viral-HCC vs. viral marker negative HCC

	**ALL HCC cases**	**Comparison of viral-HCC vs. viral marker negative HCC**		
	**n = (645)**			
	**Mean ± SD/n (%)**	**Viral HCC (n = 546)**	**Viral marker negative HCC(n = 99)**	**P value**
**Duration between diagnosis of cirrhosis & HCC(months)**				
00	291(45.1)	226(41.4)	65(65.7)	**<0.001**
1-55	243(37.7)	219(40.1)	24(24.2)	
>55	111(17.2)	101(18.5)	10(10.1)	
**Mode of diagnosis**				
On screening	53(8.2)	52(9.5)	1(1.0)	**0.001**
Incidental	57(8.8)	44(8.1)	13(13.1)	
Symptomatic	535(82.9)	450(82.4)	85(85.9)	
**Stage of HCC**				
Non-advance	240(37.2%)	222(40.7)	18(18.2)	**<0.001**
Advance	405(62.8%)	324(59.3)	81(81.8)	
**Macroscopic types**				
Solitary	246(38.1)	213(39.0)	33(33.3)	**0.04**
Paucifical (≤ 3 nodules)	259(40.2)	223(40.8)	36(36.4)	
Multifocal(>3 nodules)	96(14.9)	79(14.5)	17(17.2)	
Massive/infiltrative	44(6.8%)	31(5.7)	13(13.1)	
**Maximum tumor size (cm)**				
< 5	359(55.65)	326(59.7)	33(33.3)	**<0.001**
5-10	213(33.02)	173(31.7)	40(40.4)	
>10	73(11.31)	47(8.6)	26(26.3)	
**Hepatic lobes**				
Left	81(12.6)	69(12.6)	12(12.1)	0.98
Right	387(60)	327(59.9)	60(60.6)	
Both	177(27.4)	150(27.5)	27(27.3)	
**PVT**				
No	429(66.5)	359(65.8)	70(70.7)	0.33
Yes	216(33.5)	187(34.2)	29(29.3)	
**Extra hepatic spread**				
No	560(86.8)	479(87.7)	81(81.8)	0.12
Yes	85(13.2)	67(12.3)	18(18.2)	

On univariate logistic regression analysis older age, male gender, concomitant DM & HTN, hypersplenism, PSE, HRS, elevated ALT, platelets counts, duration between diagnosis of cirrhosis and HCC, modality of diagnosis, stage, size and macroscopic types of HCC, presence of extra hepatic spread were found as significant independent prognostic factors (Table 3). However, when adjusted for other independent prognostic factors; larger tumor size (p <0.001), shorter duration between diagnosis of cirrhosis and HCC (p 0.03), concomitant DM (<0.001) were found significant factors associated with viral marker negative HCC (Table 4).

**Table 3:** Univariate analysis for prognostic factors of viral-HCC vs. viral marker negative HCC

**Variables**	**OR(95% CI)**	**P value**	**Variables**	**OR(95% CI)**	**P value**
**Age(years)**	1.026(1.01-1.05)	0.01	**Hepatopulmonary Syndrome**		
**Gender**			No	1.0	0.19
Female	1.0	0.34	Yes	1.62(0.80-3.29)	
Male	1.26(0.77-2.05)		**Total Bilirubin(mg/dl)**		
**BMI** (Kg/m^2^)			<2	1.0	0.48
18-22.9	1.0	0.24	2-3	0.93(0.51-1.67)	
<18	2.08(0.90-4.77)		>3	1.29(0.79-2.09)	
23-24.9	1.34(0.74-2.43)		**Albumin(mg/dl)**		
≥25	0.98(0.56-1.69)		>3.5	1.0	0.73
**Diabetes Mellitus**			2.8-3.5	0.73(0.32-1.62)	
No	1.0	0.02	<2.8	0.74(0.35-1.56)	
Yes	1.64(1.07-2.53)		**ALT (IU/ml)**		
**Hypertension**			Normal	1.0	0.19
No	1.0	0.11	Elevated	0.58(0.37-0.91)	
Yes	1.42(0.92-2.21)		**Prothrombin time(second)**		
**Dyslipidemia**			<3	1.0	0.14
No	1.0	0.74	3-6	0.61(0.3-1.02)	
Yes	0.74(0.17-3.50)		>6	0.91(0.53-1.55)	
**Child score**	1.02(0.93-1.12)	0.59	**Platelets counts (10**^**9**^**/L)**	1.01(1.0-1.01)	<0.001
**Child class**			**AFP(IU/ml)**		
A	1.0	0.50	≤200	1.0	0.53
B	1.02(0.50-2.11)		>200	0.86(0.56-1.34)	
C	1.32(0.65-2.67)		**Duration between diagnosis of cirrhosis**		
**Okuda class**			00	2.90(1.43-5.88)	<0.001
I	1.0	0.97	1-55	1.10(0.51-2.40)	
II	0.99(0.52-1.87)		>55	1.0	
III	1.15(0.51-2.15)		**Mode of diagnosis**		
**Complication of CLD**			On screening	1.0	0.001
No	1.0	0.39	Incidental	15.36(1.93-122.14)	
Yes	1.24(0.74-2.07)		Symptomatic	9.82(1.34-72.01)	
**Hyperspleenism**			**Stage of HCC**		
No	1.0	0.14	Non-Advance	1.0	<0.001
Yes	0.72(0.46-1.11)		Advance	3.08(1.79-5.28)	
**Ascites**			**Macroscopic types of HCC**		
No	1.0	0.84	Solitary	1.0	0.04
Yes	0.95(0.60-1.51)		Paucifocal (≤ 3 nodules)	1.04(0.62-1.73)	
**PSE**			Multifocal(>3 nodules)	1.38(0.73-2.63)	
No	1.0	0.23	Massive/infiltrative	2.7(1.28-5.69)	
Yes	1.31(0.84-2.03)		**Maximum tumor size(cm)**		
**Esophageal Varices**			< 5	1.0	<0.001
No	1.0		5-10	2.08(1.39-3.75)	
Yes	0.84(0.55-1.29)	0.44	>10	5.46(3.01-9.93)	
**Upper GI bleed**			**Hepatic Lobes**		
No	1.0		Left	1.0	0.98
Yes	0.90(0.58-1.40)	0.64	Right	1.05(0.53-2.06)	
**Hepatohydrothorax**			Both	1.03(0.49-2.16)	
No	1.0	0.43	**Porto Venous Thrombosis**		
Yes	1.32(0.66-2.65)		No	1.0	0.33
**Hepatorenal Syndrome**			Yes	1.25(0.788-2.00)	
			**Extra hepatic spread**		
No	1.0	0.50	No	1.0	0.12
Yes	1.18(0.72-1.94)		Yes	1.58(0.89-2.81)	

**Table 4:** Multivariable logistic regression analysis for prognostic factors of viral-HCC vs. viral marker negative HCC

	**OR(95% CI)**	**P value**
**Maximum tumor size(cm)**		
< 5	1.0	<0.001
5-10	2.28(1.37-3.81)	
>10	5.30(2.82-9.96)	
**Duration between diagnosis of cirrhosis and HCC (months)**		
00	2.56(1.23-5.34)	0.02
1-55	1.14(0.51-2.52)	
>55	1.0	
**Diabetes Mellitus**		
No	1.0	<0.00
Yes	1.94(1.23-3.07)	

### Comparison of viral marker negative HCC with HCC due to hepatitis B, C, or combination of viruses

Table 5 depicts, unadjusted odds ratios along with 95% CI. Analysis showed that with HCV as reference, the male gender was significantly associated with HBV, co-infections and viral marker negative HCC. The tumor size of 5–10 cms, was also significantly associated with HCC due to hepatitis B and with viral maker negative HCC versus HCV. However, variables like extra hepatic spread, complications of cirrhosis, Child’s score, Okuda score, were not found significant on univariate analysis.

**Table 5:** Comparison of viral marker negative HCC with HCV-HCC, HBV-HCC, combination-HCC

	**Percentages**				**Unadjusted OR(95% CI)**			**Adjusted OR(95% CI)**		
**Variable**	**HCV**	**HBV**	**Combination**	**NBN**	**HBV**	**Combination**	**NBNC**	**HBV**	**NBNC**	**Combination**
**Gender**										
Female	35.6	17.6	17.9	25.3	1			1		
Male	64.4	82.4	82.1	74.7	2.5 (1.5-4.3)	2.5 (1.2-5.1)	1.6 (0.9-2.6)	2.3 (1.3-3.8)	1.5 (0.8-2.5)	2.6 (1.2-5.3)
**Diagnosis of HCC**										
On screening	11.9	2.5	1.9	1.0	1			1		
incidental	8.1	9.2	5.4	13.1	5.3 (1.3-20.9)	0.88 (0.19-3.96)	19.06 (2.3-153.5)	3.4 (0.8-13.9)	10.1 (1.2-84.7)	0.8 (0.17-3.8)
symptomatic	80.1	88.2	85.7	85.9	5.1 (1.5-17.0)	1.4 (0.53-3.76)	12.5 (1.7-92.7)	4.1 (1.2-13.9)	8.1 (1.1-60.3)	1.4 (0.5-3.9)
**BMI (Kg/m**^**2**^**)**										
18-22.9	26.7	35.3	26.8	25.3	1			1		
<18	3.8	11.8	3.6	10.1	2.3 (1.03-2.37)	0.94 (0.19-4.56)	2.8 (1.12-7.11)	2.8 (1.2-6.6)	3.1 (1.1-8.3)	1.1 (0.2-5.0)
23-24.9	22.9	21.0	26.8	27.3	0.69 (0.39-1.23)	1.16 (0.53-2.52)	1.25 (0.53-2.52)	0.7 (0.3-1.3)	1.3 (0.6-2.4)	1.1 (0.5-2.4)
≥ 25	46.6	31.9	42.9	37.4	0.51 (0.31-0.85)	0.91 (0.45-1.82)	0.84 (0.48-1.48)	0.6 (0.3-1.1)	0.9 (0.5-1.7)	1.0 (0.5-2.0)
**Maximum diameter (cm)**										
<5	62.5	49.6	62.5	33.3	1			1		
5-10	29.1	39.5	32.1	40.5	1.7 (1.09-2.6)	1.1 (0.59-2.0)	2.6 (1.5-4.3)	1.5 (0.9-2.5)	2.3 (1.3-4.0)	0.9 (0.5-1.7)
>10	8.4	10.9	5.4	26.3	1.64 (0.8-3.3)	0.64 (0.18-2.2)	5.8 (3.1-11.1)	1.0 (0.5-2.2)	4.0 (2.0-7.9)	0.5 (0.1-1.7)
**Duration between diagnosis of CLD and HCC**										
>55	22.1	6.7	19.6	10.1	1			1		
zero	37.5	52.1	44.6	65.7	4.5 (2.0-10.0)	1.3 (0.62-2.8)	3.8 (1.8-7.8)	3.7 (1.6-8.4)	3.0 (1.4-6.5)	1.1 (0.4-2.4)
1-55	40.4	41.2	35.7	24.2	3.3 (1.5-7.4)	0.9 (0.4-2.1)	1.3 (0.5-2.8)	2.6 (1.1-6.1)	1.2 (0.5-2.8)	0.7 (0.3-1.7)
**Diabetes**										
No	55.8	72.3	78.6	49.5	1			1		
Yes	44.2	27.7	21.4	50.5	0.48 (0.30-0.76)	0.34 (0.17-0.67)	1.2 (0.8-2.0)	0.5 (0.3-0.9)	1.6 (0.1-2.5)	0.3 (0.2-0.6)

The multivariable analysis showed that, the males had a greater risk of developing HCC due to hepatitis B, co-infections due to HBV/HCV/HDV (mOR 2.3, 2.6, 1.5 respectively) as compared to females. The risk of being diagnosed when symptomatic was 4.1 times and 8.1 times higher in case of HBV related HCC and viral marker negative HCC respectively (mOR 4.1 and 8.1 respectively) as compared to HCV related HCC. Tumor size >5 cm, was only found to be significantly associated with viral marker negative HCC. Likewise, the risk of being diagnosed to have underlying cirrhosis and HCC simultaneously at presentation was higher in case of HBV and viral marker negative HCC (mOR 3.7 and 3.0 respectively). Moreover, BMI <18 was significantly associated with HCC due to hepatitis B and viral marker negative HCC (mOR 2.8 and 3.1). This could be attributed to malnutrition and weight loss due to more advanced HCC in these cases. However, HBV and HCV were less likely when BMI was ≥ 25 (mOR 0.6 and 0.9). Furthermore, the odd of having diabetes was 1.6 times higher in NBNC compared to HCV related HCC.

### Applicability of treatment for HCC

According to the Barcelona Clinic Liver Cancer (BCLC) staging classification 9(1.4%) patients had very early stage (0) HCC; 2(0.3%) of them underwent liver transplantation and 7(1.1%) had tumor resection. Moreover, 9(1.4%) patients had early stage HCC (A) and they had percutaneous ethanol ablation (PEA). However none HCC patients in BCLC stage 0 or stage A belonged to viral marker negative HCC group. This was due to the fact that majority of viral marker negative HCC patients (81.8%) had advanced HCC on presentation.

A total of 260(40.3) patients had BCLC-Intermediate stage (B) HCC out of which Transarterial chemoembolization (TACE) was done in 247 (38.3%) cases; out of these 247 cases only 13.37% had viral marker negative HCC. BCLC-advanced stage (C) HCC was found in 8(1.2%) HCC cases (six had viral- HCC and two had non-viral HCC) where either Gemcitabine or Sorafenib was prescribed as chemotherapeutic agent. Overall 372 (57.7%) patients were treated conservatively due to underlying advanced cirrhosis/HCC (BCLS stage End stage (D)) or contraindication for HCC treatment. Hence, the applicability of treatment for HCC was limited for viral marker negative HCCs due to underlying advanced cirrhosis and HCC at presentation.

## Discussion

Hepatocellular carcinoma is a major concern globally and to the best of our knowledge, this is the largest study from Pakistan carried out on HCC ever. We analyzed the trends and the clinico-pathological characteristics of HCC; comparing them between HCC related to viral hepatitis and viral marker negative HCC; in our native South Asian population of Pakistan. A consistent rise in proportion of all, including viral marker negative HCC has been observed and a higher proportion of patients (15.3%) had HCC negative for serological markers of hepatitis B and C. Being not under proper surveillance for HCC, most of the viral marker negative HCCs were diagnosed when advanced and symptomatic. Moreover, significant association was found between DM and viral marker negative HCC. The variation from HBV to HCV as a leading cause of HCC has also been documented in many countries of South-east Asia [2]. Also, a gradual rise in NBNC-HCC has been observed in recent years. In a recent study from Japan, out of 1374 HCC cases 15% were negative for HBsAg and antibody to hepatitis C virus and a significant association of Diabetes, HTN and dyslipidemia was established with NBNC-HCC [28]. In few small studies from Pakistan the reported proportions of viral marker negative HCC ranged between 13.6%-16% [29,30]. Our results are consistent with the studies reported from the region with a potential strength of greater generalizability due to a larger sample size. However, the higher prevalence of viral marker negative HCC has been found in certain studies conducted in Western populations [31-33] which could be due to the existing difference in exposure to the various etiological factors leading to HCC.

HCCs at the early-stage are usually asymptomatic, and are mostly discovered during periodic surveillance. It being diagnosed when symptomatic, increases the risk of having more advance disease when caught, with worse prognostic factors [1], as seen in our study. In 65.7% of non-viral HCC and 41.4% of viral HCC diagnosis of cirrhosis and HCC were made simultaneously. Our results were consistent with the study conducted by Toyoda and colleagues [3] where out of 1152 Japans patients with HCC, 10.3% had NBNC-HCC. NBNC-HCCs were more advanced, larger in size and associated with more frequent vascular invasion and extrahepatic spread at the time of diagnosis. Moreover, almost half of NBNC-HCC cases were not under surveillance as compared to viral HCC (42 vs. 22.9%, p <0.0001). In another study of 624 patients from Japan [34] viral marker negative HCCs were found to be associated with poor prognosis. Furthermore, male gender, Child’s core, TNM stage were the significant prognostic factors in that study. In our study we observed shorter time interval between the diagnosis of cirrhosis and HCC, more advanced, larger, multifocal, massive or infiltrative tumors in case of viral marker negative HCC as compared to viral HCC. This was probably due to the fact that most of patients with non-viral HCC were not symptomatic and were not under surveillance for HCC. While due to better awareness about the risk of cirrhosis and HCC associated with hepatitis B and C, most of such patients were diagnosed to have cirrhosis earlier than HCC diagnosed. Thus, due to lack of surveillance, most of NBNC-HCC were diagnosed when symptomatic leading to a later diagnosis. The patients with viral marker negative HCCs tended to be older and diabetic as compared to the viral HCC. However, no significant difference was found in distribution of gender, BMI, stage of underlying chronic liver disease in our study.

Owing to the involvement of distinct oncogenic pathways in the development of HCC, it is well expected that difference exists in the natural course of HCC and prognosis due to different hepatotropic viruses and other non-viral etiologies [3,13]. In a study by Dohmen and colleagues [5] younger age, HCC >3 cm in size, multiple lesions, PVT were strongly associated with HBV related HCC as compared to HCC due to HCV and non-viral HCC. Poor survival was found for HBV related HCC and NBNC-HCC had a longer survival rate. Similar results were found in another study of 2,542 HCC patients from Japan [13]. Trevisani and colleagues compared the HBV, HCV, alcohol and multietiology (combination of different hepatotropic viruses with and without alcohol) related HCC among 742 Italian HCC patients [35]. In their study, infiltrative HCCs were found more common in HBV-HCC group; and CLIP stage 3 was more frequent in HBV and multietiology group. However, the influence of etiology of gross pathology was not significant. In our study, regardless of underlying etiology a significant association of male gender was found with HCC. HBV-HCC and viral marker negative HCCs were found to be more advanced and aggressive at the time of presentation. Most of them were more symptomatic, larger in size and were diagnosed simultaneously with the underlying cirrhosis. These findings support the greater carcinogenic activity of hepatitis B and non-viral etiologies. We did not find any strong associations in clinico-pathological characteristics and stage of HCC in co-infection group. This could be due to small sample of patients in co-infection group. Diabetes had a significant association with viral marker negative HCC which could be due to underlying non-alcoholic steatohepatitis.

The studies describing the characteristics and prognosis of viral marker negative HCC are limited and most involved the patients who underwent hepatic resection, selected as the study population; hence, might not truly reflect the entire population of viral marker negative HCC [5,36,37]. Differences exist in exposure to the various etiological factors for HCC and host factors in Asian population as compared to West. However, limited data is available from South-Asia. Our study comprises a large sample of patients referred from all over the country and reflects well the scenario of HCC in patients with cirrhosis belongs to a South Asian community. However, our study has certain limitations. It is a single center based, cross sectional study; therefore the true cause and effect relationship could not be ascertained. Furthermore, for the analysis of HBV infection, HBsAg was tested while HBeAg, Anti-HBe, Anti-HBc, Anti-HBs, and HBV DNA were not checked. In majority of cases further work to evaluate HBV infection and viral replication was not offered by their primary physicians. Hence, only serological marker for HBV exposure available was HBsAg. Moreover, due to its cross sectional design despite large sample there are certain biases. Most of our patients with unresectable HCC were diagnosed incidentally or when they were symptomatic. Hence, the true interval between disease progression from exposure to development of HCC could not be established. For consistency of results, better exploration of natural history, and prognosis of HCC in the South-Asian population, population based studies are needed.

## Conclusion

To conclude, the burden of hepatocellular carcinoma is on the rise amongst native South Asian Pakistani population and viral marker negative HCC is not uncommon here. Patients with viral marker negative HCC tend not to be under surveillance as compared to viral-HCC and are diagnosed mostly with a more advanced disease when symptomatic. Hence, viral marker negative HCC (vs. viral-HCC) were more advanced, larger in size, mostly multifocal, massive or infiltrative at the time of presentation. Moreover, viral marker negative and HBV related HCC were more symptomatic, advanced with larger tumor size on presentation as compared to HCC due to HCV or co-infection with HBV/HCV/HDV. Diabetes Mellitus was found to be a significant factor associated with viral marker negative HCC. We believe that it is of great importance that ample light be shed on the need for strategies that can help in implementation of proper screening and surveillance of HCC for all cirrhotic patients especially when cirrhosis is due to non-viral etiologies.

## Abbreviations

HCC: Hepatocellular carcinoma; HBV: Hepatitis B virus; HCV: Hepatitis C virus; NASH: Nonalcoholic steatohepatitis; HDV: Hepatitis D virus; AKUH: Aga Khan University hospital; ICD: International classification of diseases; AFP: Alfa fetoprotein; CT: Computerized tomography; MRI: Magnetic resonance imaging; ALT: Alanine aminotransferase; HBsAg: Hepatitis B surface Antigen; Anti-HCV: Anti-HCV antibody); DM: Diabetes Mellitus; HTN: Hypertension; BMI: Body mass index; CTP: Child-turcotte-pugh score; mOR: Multinomial odds ratios; OR: Odds ratio.

## Competing interests

The authors declared that they have no competing interests.

## Authors’ contributions

ASB conceived the study question, conducted the study, performed the statistical analysis and wrote manuscript. SH, AAW, TUH and WJ participated in the design of the study and reviewed manuscript. BA helped in statistical analysis and reviewed the manuscript. MG, AI, OF collected and entered the data in SPSS file. All authors read and approved the final manuscript.
